# Tetramodal Chemical Imaging Delineates the Lipid–Amyloid Peptide Interplay at Single Plaques in Transgenic Alzheimer’s Disease Models

**DOI:** 10.1021/acs.analchem.2c05302

**Published:** 2023-03-01

**Authors:** Junyue Ge, Srinivas Koutarapu, Durga Jha, Maciej Dulewicz, Henrik Zetterberg, Kaj Blennow, Jörg Hanrieder

**Affiliations:** † Department of Psychiatry and Neurochemistry, Sahlgrenska Academy at the University of Gothenburg, Mölndal Hospital, House V3, SE-431 80 Mölndal, Sweden; ‡ Clinical Neurochemistry Laboratory, Sahlgrenska University Hospital, Mölndal Hospital, House V3, SE-431 80 Mölndal, Sweden; § Department of Neurodegenerative Disease, Queen Square Institute of Neurology, University College London, London WC1N 3BG, United Kingdom; ∥ UK Dementia Research Institute at University College London, Queen Square, London WC1N 3BG, United Kingdom; ⊥ Hong Kong Center for Neurodegenerative Diseases, Hong Kong 1512-1518, China; # Wisconsin Alzheimer’s Disease Research Center, University of Wisconsin School of Medicine and Public Health, University of Wisconsin-Madison, Madison, Wisconsin 53726, United States

## Abstract

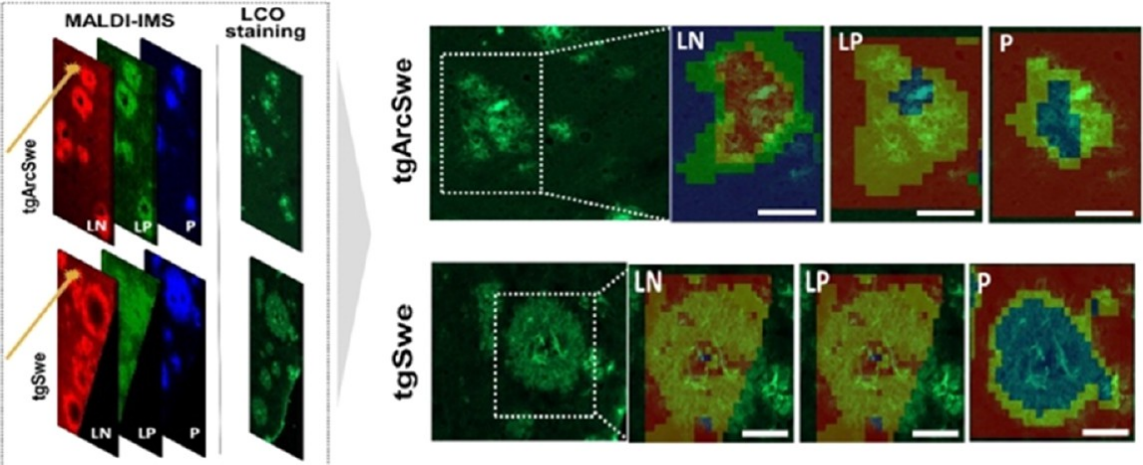

Beta-amyloid (Aβ) plaque pathology is one of the most prominent histopathological feature of Alzheimer’s disease (AD). The exact pathogenic mechanisms linking Aβ to AD pathogenesis remain however not fully understood. Recent advances in amyloid-targeting pharmacotherapies highlight the critical relevance of Aβ aggregation for understanding the molecular basis of AD pathogenesis. We developed a novel, integrated, tetramodal chemical imaging paradigm for acquisition of trimodal mass spectrometry imaging (MSI) and interlaced fluorescent microscopy from a single tissue section. We used this approach to comprehensively investigate lipid–Aβ correlates at single plaques in two different mouse models of AD (tgAPP^Swe^ and tgAPP^ArcSwe^) with varying degrees of intrinsic properties affecting amyloid aggregation. Integration of the multimodal imaging data and multivariate data analysis identified characteristic patterns of plaque-associated lipid- and peptide localizations across both mouse models. Correlative fluorescence microscopy using structure-sensitive amyloid probes identified intra-plaque structure-specific lipid- and Aβ patterns, including Aβ 1–40 and Aβ 1–42 along with gangliosides (GM), phosphoinositols (PI), conjugated ceramides (CerP and PE-Cer), and lysophospholipids (LPC, LPA, and LPI). Single plaque correlation analysis across all modalities further revealed how these distinct lipid species were associated with Aβ peptide deposition across plaque heterogeneity, indicating different roles for those lipids in plaque growth and amyloid fibrillation, respectively. Here, conjugated ceramide species correlated with Aβ core formation indicating their involvement in initial plaque seeding or amyloid maturation. In contrast, LPI and PI were solely correlated with general plaque growth. In addition, GM1 and LPC correlated with continuous Aβ deposition and maturation. The results highlight the potential of this comprehensive multimodal imaging approach and implement distinct lipids in amyloidogenic proteinopathy.

## Introduction

Alzheimer’s disease (AD) is the most common form of dementia affecting around 1 in 8 over the age of 65.^1^ The prevalence of the disease is increasing with age and hence AD is a major health burden. This is further aggravated by a general increase in the average age of the world’s population and most significantly the absence of any curative treatment, so far. AD pathology is characterized by the formation of extracellular plaques consisting of amyloid β (Aβ) and neurofibrillary tangles, consisting of hyperphosphorylated tau protein.^2^ The persisting hypothesis of AD pathogenesis postulates that amyloid upon aggregation triggers a cascade of pathogenic, neurotoxic events downstream.^3,4^ While the exact mechanisms linking Aβ aggregation to AD pathogenesis remain not fully understood, the amyloid hypothesis is significantly supported by recent phase 3 results for the amyloid-targeting antibody lecanemab.^5^ Importantly, there is a huge variety among Aβ aggregation intermediates (monomers, oligomers, protofibrils, and fibrillar Aβ) that have been implicated with Aβ pathogenicity at varying degrees.^6−8^ Moreover, beyond intrinsic factors (Aβ truncation and conformation), extrinsic factors such as neuronal lipids have been implicated in Aβ pathology. This is supported by the fact that the apolipoprotein E (*APOE*) ε4 allele, which encodes a lipid transporter protein, increases the risk of sporadic AD several-fold.^9^

In addition, genome-wide association studies identified mutations in *ABCA*7, a phospholipid transporter, and within the lipid sensing microglia surface receptors *TREM*2 as genetic risk factors of AD.^10^ This highlights the need for analytical tools that deliver the necessary chemical specificity and sensitivity to delineate different plaque associated biochemical species and contextualize those with chemical properties of Aβ aggregation *in situ*. In particular, mass spectrometry imaging (MSI) has been demonstrated to be a powerful tool for interrogating amyloid–plaque pathology in AD brain tissue both in patients and mouse models.^11−13^ Specifically, our group has been introducing correlative MSI methods to acquire multimodal lipid and peptide imaging data from the same section in AD brain tissues.^14−16^

Herein, we set out to make use of these developments. Specifically, we implemented trimodal matrix-assisted laser desorption/ionization (MALDI) MSI^14^ along with interlaced, fluorescent microscopy. We then used this tetramodal imaging paradigm to investigate Aβ pathology-associated distribution patterns of lipids and proteins in transgenic AD mice. As a further novelty of this approach, we combined and comprehensively interrogated the correlative MSI data obtained from single Aβ plaque regions of interest (ROI) acquired from the same tissue section using multivariate analysis. Using this correlative imaging and image analysis approach, we investigated lipid and peptide differences in polymorphic plaque pathology across two genetic models with varying degrees of cerebral amyloidosis (tgSwe and tgArcSwe). Here, the additional arctic mutation in tgArcSwe mice modifies the chemical properties of Aβ leading to accelerated Aβ aggregation.^17^ We hypothesize that comparing these models will provide clues on how lipid–amyloid interaction is related to accelerated plaque formation, -growth, and amyloid fibrillation. We further present novel approaches to interrogate the comprehensive multimodal imaging signatures from single plaques using multivariate analyses. This allowed us to identify distinct plaque phenotypes and their associated chemical architecture. By contextualizing those signatures with the genetic background and associated Aβ properties of different APP mouse models provided insight into the potential role of lipids in amyloid–plaque formation, plaque growth, and amyloid peptide fibrillation.

## Experimental Section

### Chemicals and Reagents

All chemicals and solvents were used without further purification: acetic acid (Cat.#: 64197, VWR Chemicals, Radnor, PA, USA), acetonitrile (ACN, Cat.#: 75058, Fisher Scientific, Waltham, MA, USA), chloroform (Cat.#: 67663, RCILabScan, Bangkok, Thailand), 1,5-diaminonaphthalene (DAN, Cat.#: 56451, Sigma Aldrich, St.Louis, MO, USA), 2′,5′-dihydroxyacetophenone (DHA, Cat.#: D107603, Sigma Aldrich), ethanol (Cat.#: V002075; Sigma Aldrich), formic acid (FA, Cat.#: 56302, Honeywell), and trifluoroacetic acid (TFA, Cat.#: 40967; Honeywell, Charlotte, NC, USA). Luminescent conjugated oligothiophene (LCO) tetramer formyl thiophene acetic acid (q-FTAA) and heptamer formyl thiophene acetic acid (h-FTAA) were obtained from Prof. Peter Nilsson, Department of Chemistry, Linköping University. Water was obtained from a Synergy UV water purification system (Milli-Q, Merck Millipore, Darmstadt, Germany).

### Animals and Tissue Collection

For this project, we studied two transgenic AD mouse models carrying mutations of human APP. Here, 18mo animals carrying the Swedish APP mutation (tgSwe, *n* = 3) and 18mo, bigenic mice carrying the Arctic and Swedish mutation of APP (tgArcSwe, *n* = 3) were investigated. Animal procedures were approved by an ethical committee and performed in compliance with national and local animal care and use guidelines (DNr #C17/14 at Uppsala University). The mice were reared ad libitum at an animal facility at Uppsala University under a 12/12 light cycle. Fresh brain tissue samples were obtained from 18 to 19-month-old female tgAPP mice. Animals were anesthetized with isoflurane and sacrificed by decapitation. The brains were dissected quickly with less than 3 min post-mortem delay and frozen on dry ice. Frozen tissue sections (12 μm) were cut using a cryostat microtome (Leica CM 1520, Leica Biosystems, Nussloch, Germany) at −18 °C, and collected on indium tin oxide (ITO) conductive glass slides (Cat.#: 237001; Bruker Daltonics, Bremen, Germany) and stored at −80 °C. Prior to analysis, tissue sections were thawed under vacuum for 1 h.

### Sample Preparation

The here employed trimodal MSI approach included the sequential acquisition of (1) dual-polarity lipid imaging data, followed by (2) matrix removal and fluorescent amyloid imaging, and final (3) tissue preparation and matrix application for amyloid peptide imaging. For MALDI MSI of lipids, the 1,5-diaminonaphthalene (DAN, Cat.#: 56451, Sigma Aldrich) matrix was applied to defrosted tissue sections using a TM sprayer (HTX Technologies, Carrboro, North Carolina) combined with an HPLC pump (Dionex P-580, Sunnyvale, California). Before spraying, the solvent pump was purged with 70% aqueous acetonitrile (ACN_aq_) at 300 μL/min for 5 min followed by manual rinsing of matrix loading loop using a syringe. A matrix solution containing 20 mg/mL DAN in 70% ACN_aq_ was sprayed onto the tissue sections with the following instrumental parameters: nitrogen flow (10 psi), spray temperature (75 °C), nozzle height (40 mm), five passes with offsets and rotations, spray velocity (1250 mm/min), and isocratic flow of 50 μL/min using 70% ACN_aq_ as the pushing solvent.

After lipid analysis in negative ion mode, tissue sections were resprayed with three passes of the DAN matrix for lipid analysis in the positive ion mode.

Fluorescent microscopy was performed between MALDI MSI lipid analyses and peptide analysis on the same tissue section as described further below.

Following fluorescent imaging, the tissue sections were exposed to vapors of concentrated formic acid for 25 min for Aβ peptide signal enhancement, as previously described in detail.^12,15^ For MALDI MSI of amyloid peptides, 2′,5′-dihydroxyacetophenone (DHA, Cat.#: D107603, Sigma Aldrich) was used as the matrix compound and applied using the TM Sprayer (HTX Technologies). A matrix solution of 15 mg/mL DHA in 70% ACN/2% CH_3_COOH/2% TFA was sprayed onto the tissue sections using the following instrumental parameters: nitrogen flow (10 psi), spray temperature (75 °C), nozzle height (40 mm), eight passes with offsets and rotations, and spray velocity (1000 mm/min), and isocratic flow of 100 μL/min using 70% ACN as the pushing solvent.

### MALDI MS Imaging

MALDI MSI was performed on the frontal cortex of the mouse brain section using a Bruker rapifleX TissueTyper TOF/TOF mass spectrometer (Bruker Daltonics). Lipid analyses were performed in both positive- and negative ionization modes over a mass range of 400–2500 Da. Acquisition was performed in reflector mode at 10 μm spatial resolution (setting “single”), with a laser frequency of 10 kHz, and 50 shots per pixel. External calibration was performed using peptide calibration standard I (Bruker Daltonics) spotted adjacent to the tissue sections.

Peptide MSI data were acquired at 10 μm spatial resolution, at a laser pulse frequency of 10 kHz with 200 shots collected per pixel. Data were acquired in linear positive mode for a mass range of 1500–6000 Da (mass resolution: *m*/Δ*m* = 1000 (FWHM) at *m*/*z* 4515). Pre-acquisition calibration of the system was performed using a combination of peptide calibration standard II and protein calibration standard I, to ensure calibration over the entire range of potential Aβ species.

### Fluorescent Amyloid Imaging

The exact preparation workflow for tetramodal imaging comprised (i) dual polarity lipid MALDI MSI (1. negative ion mde, 2. positive ion mode) with matrix re-application after the first acquisition, (ii) removal of the matrix by tissue washing, (iii) LCO fluorescence staining, (iv) fluorescent image acquisition, and (v) peptide MALDI MSI.

Fluorescent imaging was performed after the MALDI MSI lipid analyses and prior to MALDI MSI peptide analysis, all on the same tissue section. For this, the remaining matrix after MALDI MSI (positive ion mode) lipid analysis was removed prior to fluorescent staining by sequential washes of 95% EtOH for 30 s, 95% EtOH for 60 s, 70% EtOH for 30 s, Carnoy’s solvent (60% EtOH, 30% chloroform, 10% acetic acid) for 90 s, and 95% EtOH for 10 s, 0.2% TFA for 60 s, and 95% EtOH for 10s. The tissue sections were then incubated in the dark at room temperature (23 °C) for 25 min with a combination of two luminescent conjugated oligothiophenes (LCOs) amyloid probes: tetrameric formyl thiophene acetic acid (q-FTAA, 3 μM) and heptameric formyl thiophene acetic acid (h-FTAA, 3 μM). After staining, the tissue sections were washed 3 times in water for 2 min each and dried at room temperature (23 °C) for a minimum of 30 min before image acquisition.

Fluorescent microscopy of LCO-stained brain sections was performed using an automatic widefield microscope (Axio Observer Z1, Zeiss, Germany). Large multi-channel tile scans were captured using EGFP filter sets. All of the images were captured using a Plan-Apochromat 20×/0.8 DIC air objective lens.

### Data Analysis

All lipid MS imaging data were externally calibrated using the batch-processing function in Flex Analysis (v 4.0 Bruker Daltonics). Image analysis of MSI data was performed in SCiLS (v 2021c, Bruker Daltonics, Bremen, Germany). For image segmentation, the imaging data were evaluated in SCiLS using the corresponding pipeline. Regions of interest (ROIs) were identified by bisecting *k*-mean clustering-based image segmentation. Total ion current normalized average spectra of the annotated ROIs were exported as csv file in SCiLS. This was followed by binning analysis for data reduction and introduction of a common mass list for all MS spectral data. For this, all ROI data were imported into Origin (v 8.1 OriginLab, Northampton, Massachusetts) and peaks and peak widths were detected on average spectra of each ROI using the implemented peak analyzer function. The determined bin borders for peak integration were exported as a tab-delimited text file. The bin borders were used for area under curve peak integration within each bin (peak-bin) of all individual ROI average spectra using an in-house developed R script.

Multivariate analyses of ROI MSI data were performed using MetaboAnalyst 5.0 (https://www.metaboanalyst.ca). Here, all binning data were interrogated by means of principal component analysis (PCA), orthogonal projections to latent structures discriminant analysis (OPLS-DA), and Pearson correlation analysis. Subsequent univariate statistics were performed using GraphPad Prism (v.9, GraphPad, San Diego, California). Lipids and peptides were annotated by accurate mass following previous MS/MS-based identifications reported by our group^19,20^ and others.^21,22^

## Results and Discussion

### Plaque-Associated Depletion of Sulfatide Revealed by Multivariate Image Analyses in tgArcSwe and tgSwe

**Figure 1:**
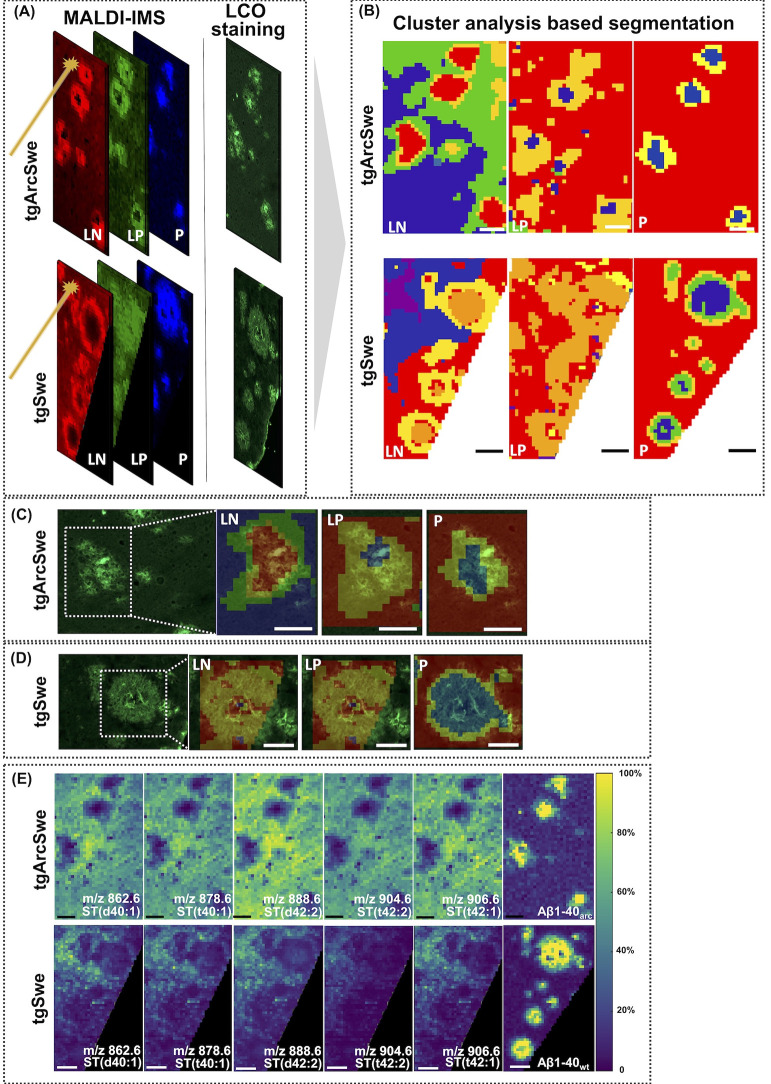
Multivariate image analyses of trimodal MSI data and correlative, aligned fluorescent data. (A) Tetramodal images in tgArcSwe and tgSwe AD mouse models. (B) Hierarchical clustering analysis (HCA) segmentation images reveal plaque features for lipid/peptide data in tgArcSwe and tgSwe AD mouse models. Scale bar: 80 μm. LN: negative ion mode lipid-, LP: positive ion mode lipid-, P: peptide-MSI; (C) Overlays of LCO-based amyloid microscopy and MSI segmentation images in the tgArcSwe AD mouse model. Scale bar: 60 μm. (D) Overlays of LOC-based amyloid microscopy and MSI segmentation images of the respective MSI modality in the tgSwe AD mouse model. Scale bar: 80 μm. (E) Ion images of sulfatides (ST) showing depletion at plaques in tgArcSwe and tgSwe AD mouse models. Black scale bar: 60 μm; white scale bar: 80 μm.

We here developed a trimodal MSI approach interfaced with correlative fluorescence microscopy to investigate the biochemical makeup of extracellular amyloid–plaque pathology in transgenic AD mouse model systems (Figure 1). We have previously reported how multimodal MSI acquisition affects both the ion signals of the subsequently acquired modality as well as tissue integrity for subsequent fluorescent microscopy.^23^

Herein, we further optimized the sample preparation for trimodal MALDI MSI acquisition, to obtain spatially well-resolved, intense ion signals while preserving the tissue morphology for fluorescence imaging. In detail, the first MS imaging run was performed in negative ion mode. This allowed to acquire lipid data with a low number of laser shots, resulting in minimized mechanical distortion and consumption of molecules on the tissue section similar to previous observations.^23^ We further optimized the sample preparation for subsequent positive lipid ion imaging by re-application of the matrix. This resulted in enhanced positive lipid ion signals (Figure S1). Following optimized dual-polarity lipid imaging, fluorescent amyloid microscopy, and peptide MSI data were acquired on the same measured region (Figure 1A).

To probe the tetramodal chemical imaging data in an unbiased way, multivariate image analyses were performed (Figure 1A). For this, MSI data were evaluated using hierarchical clustering analysis (HCA, bisecting *k*-means) for segmentation of anatomical regions of interest based on their chemical identity. Here, image segmentation identified plaque-like features both in tgArcSwe and tgSwe mouse brain tissue (Figure 1B). These features were assigned as individual regions of interest (ROI) and correlated to the processed MS data to identify the associated chemical species that allowed image segmentation of the regions. To further validate the plaque identity of the HCA-derived plaque features, we employed LCO-based fluorescent amyloid microscopy. Consequently, the segmentation map showed strong colocalization of deposit-like features with Aβ deposits identified by LCO staining (Figure 1C,D). This further allowed to annotate the degree of Aβ aggregation associated with plaque polymorphism, which prominently manifests itself as compact, mature q-FTAA-positive plaque core structures surrounded by a diffuse immature, h-FTAA-positive periphery.^12^ In the HCA segmentation results, the q-FTAA-positive plaque core was characteristically outlined by pseudoclusters in the MSI data, for plaques in both tgArcSwe and tgSwe brain tissues. The q-FTAA-positive plaque core was most prominently outlined by negative ion mode lipid- (LN) and peptide- (P) MSI data (Figure 1C,D). Interestingly, HCA segmentation of positive ion mode lipid MSI data resulted in less prominent pseudoclusters outlining the core in tgArcSwe plaques, while no core-associated pseudoclusters were observed in tgSwe plaques. This indicates that only very few phosphatidylcholine (PC) lipids, as prominently seen in positive ion mode, are associated with amyloid aggregation and only at very mature stages. Following segmentation analysis, we inspected the discriminative *m*/*z* values in the annotated ROIs obtained from the HCA segmentation results. Most prominently, this revealed plaque-associated depletion of sulfatides (ST) (Figure 1E).

Further, the data showed accumulation of Aβ peptides that were colocalized with spatial sulfatide alterations as well as the LCO positive features in fluorescence microscopy (Figure 1E). This is consistent with previous studies showing the depletion of sulfatides at amyloid plaques both in tgArcSwe and tgSwe AD mouse models.^15,16,20,24^ Sulfatides are enriched in the myelin sheath of neurons and are mainly synthesized by oligodendrocytes. ST play vital roles in the regulation of oligodendrocyte maturation and myelin formation.^25,26^ Further, sulfatide depletion was found to be associated with abnormal ApoE lipoprotein metabolism in both human AD^27^ and AD mouse models.^28,29^ This is of interest as the human *APOE* ε4 allele encoding apolipoprotein E4 is a major genetic risk factor for AD. In addition, low-density lipoprotein (LDL) receptor-related protein 1 (LRP1) has been reported to play a key role in synaptic integrity and its depletion in mice results in decreased levels of sulfatides, suggesting an implication of ST depletion in synaptic degeneration.^30−32^

### Distinct Lipid Classes and Aβ Isoforms Display Specific Accumulation Patterns across the Plaque in tgArcSwe and tgSwe AD Mouse Models

In contrast to sulfatide depletion, general plaque-associated enrichment of various lipids and Aβ peptides was observed in both tgArcSwe and tgSwe AD mice. To delineate lipid and peptide accumulation patterns across these two models, we performed multivariate statistical analysis of the correlative, trimodal MSI spectral data for the individual plaque ROI.

**Figure 2:**
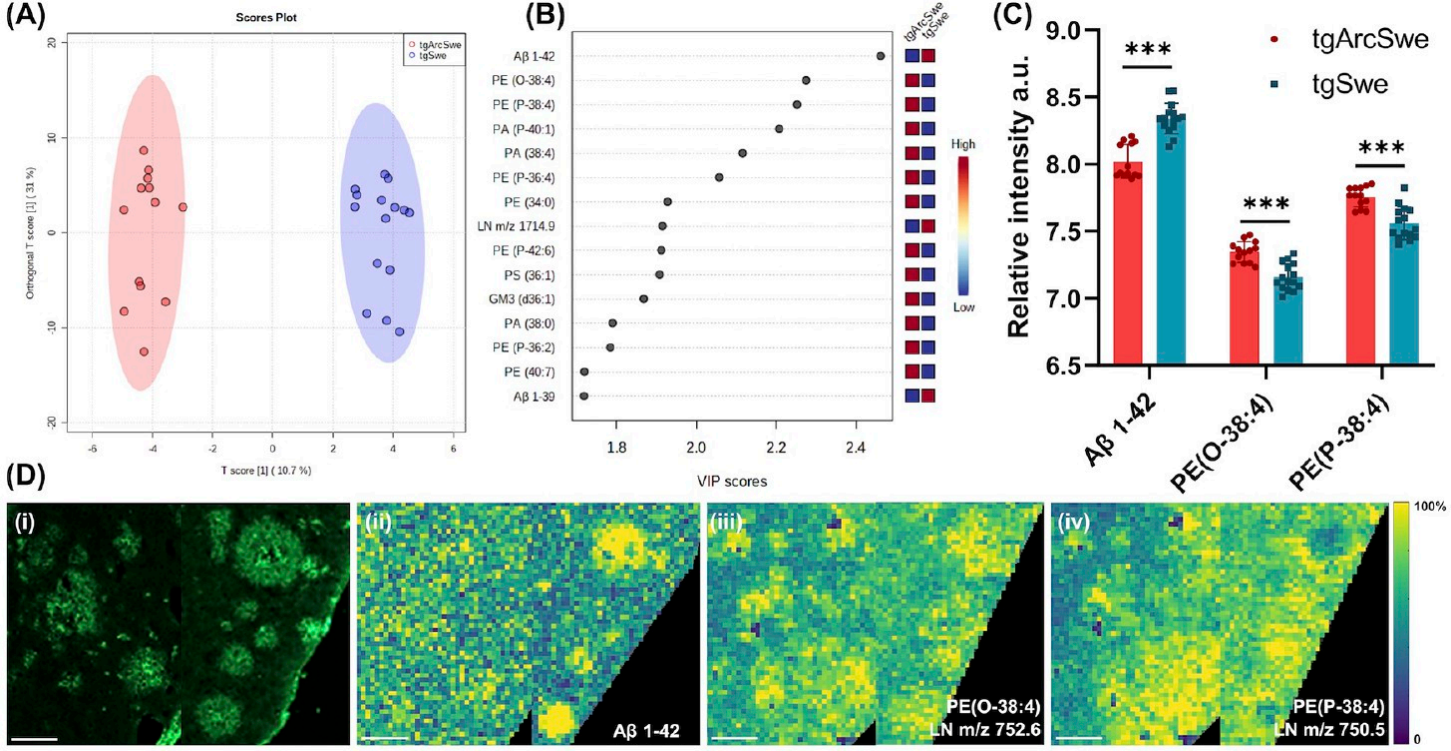
Multivariate statistical analyses of trimodal MSI data reveal different levels of lipid and peptide enrichment in whole plaques between tgArcSwe and tgSwe AD mouse models. (A) Score plot of OPLS-DA model to discriminate whole plaque ROI in tgArcSwe and tgSwe AD mouse models. (B) The top 15 discriminative lipids are ranked in descending order by variables important to projection (VIP) scores. (C) Statistical analysis of top 3 VIP differentiating the two plaque populations between tgArcSwe and tgSwe mice. (D) LCO images and single ion images of species differentiating the two plaque populations between tgArcSwe (left) and tgSwe (right). The number of animals was as follows: *n* = 3 (tgArcSwe) and *n* = 3 (tgSwe); 4–6 plaques for tgArcSwe and tgSwe mice were collected from the frontal cortex. Black scale bar: 60 μm, white scale bar: 80 μm.

**Figure 3:**
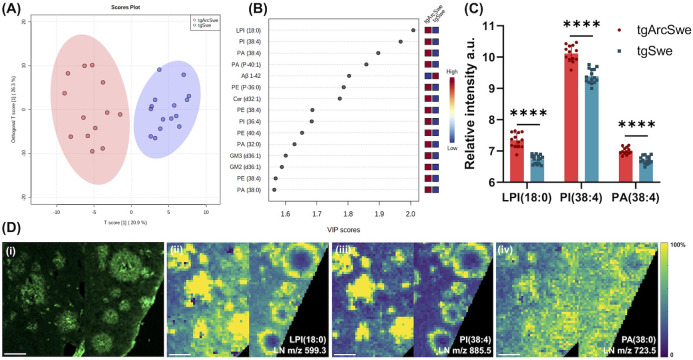
Multivariate statistical analyses of trimodal MSI data reveal different levels of lipid enrichment in the center of plaques between tgArcSwe and tgSwe mice. (A) Score plot of OPLS-DA model to discriminate the center of the plaques between tgArcSwe and tgSwe mice. (B) The top 15 discriminative species in descending order by VIP scores. (C) Statistical analysis of top 3 VIPdifferentiating the two plaque populations between tgArcSwe and tgSwe mice. (D) LCO images and single ion images of lipids differentiating plaques between tgArcSwe (left) and tgSwe (right). The number of animals was as follows: *n* = 3 (tgArcSwe) and *n* = 3 (tgSwe); 4–6 plaques for tgArcSwe and tgSwe mice were collected from the frontal cortex. Black scale bar: 60 μm, white scale bar: 80 μm.

Here, PCA and OPLS-DA were performed on both average plaque values per animal (*n* = 3/genotype) and on considering the plaques as individual observations (4–6 plaques/animal/group) to estimate the spread of the respective population in the score plots (Figures 2A, 3A, and S3). First, PCA and OPLS-DA analyses were performed on the group level of MSI data at both the whole plaque- and plaque center level (Figure S3A–F). To further identify the lipid- and peptide species differentiating the two mouse models, PCA and OPLS-DA analyses were further performed on the individual plaque level of MSI data at the whole plaque level. Here, PCA was used as the initial step to identify potential grouping patterns and estimate variation in an unbiased way (Figure S3G). OPLS-DA was then performed to identify distinct major lipid- and peptide species that differentiate the different plaque species across the two genotypes. PCA and OPLS-DA were performed on both average plaque values per animal (*n* = 3/genotype) and on considering the plaques as individual observations (4–6 plaques/animal/group) to estimate the spread of the respective population in the score plots (Figures 2A, 3A, and S3).

This revealed a clear separation of plaques between the two models based on their multidimensional, chemical identity. The most prominent chemical differences influencing group separation are reflected in the variables important for projection (VIP) scores retrieved from OPLS-DA^33^ (Figure 2B).

The results show that lipids and Aβ peptides exhibited different levels of enrichment across the whole plaque ROI between tgArcSwe and tgSwe mice. Among the top 15 VIP, 10 species were also observed among the top 15 discriminative species in the corresponding average plaque data (Figure S3C).

Here, Aβ1–42 and Aβ1–39 showed a higher level of enrichment in the whole plaque ROI in tgSwe mice than in tgArcSwe mice (Figure 2B). The other top discriminative components included phophoethanolamines (PE) and plasminogen (PE-O) lipids (PE O-38:4, PE P-38:4, PE P-36:4, PE 34:0, PE-42:6, PE P-36:2, PE 40:7), as well as phosphatidic acids (PA) (PA P-40:1, PA 38:4, PA 38:0) that all showed higher levels of enrichment at whole plaque ROI in tgArcSwe than in tgSwe mice (Figure 2C and D) (Figure S4).

It was previously reported that PE is one of the earliest phospholipids to be affected by AD pathology and its level decreases in the gray matter during the AD process.^34^

Moreover, PE (P-36:4) and PA (38:4), with high VIP scores > 1.8 and different distribution, serve as the most dominant lipid species to differentiate the two plaque populations between tgArcSwe and tgSwe mice (Figure 2D) (Figure S4). Indeed, the Arctic mutation in APP in tgArcSwe mice increases the hydrophobicity of Aβ and results in a more aggravated and accelerated Aβ plaque pathology than in tgSwe mice.^17^ The tgArcSwe model has an early-onset of plaque deposition from 3 months and more compact senile plaques, while tgSwe shows later, more graded plaque onset from approximately 9-10 months.^17^ Therefore, a higher level of lipid enrichment in the whole plaque in tgArcSwe mice likely reflects a longer deposition time of lipids at the plaque.

Furthermore, plaques in tgArcSwe mice have a more compact/aggregated homogeneous phenotype as compared to structurally more heterogeneous cored plaques in tgSwe mice that are characterized by a distinct aggregated plaque core and a diffuse periphery.^12,15^ The heterogeneous plaque pattern observed in tgSwe but not tgArcSwe can be attributed to the later, more physiological onset age for plaque deposition (9–10 months) as compared to tgArcSwe, where plaques form as early as 3 months.^17,35^

Given this difference in plaque morphology, we refined our analysis towards the center area of the plaques in tgArcSwe and tgSwe mice (Figure 3). OPLS-DA allowed discrimination between plaque center ROI from tgArcSwe and tgSwe mice (Figure 3A). These analyses revealed enrichment of glycerophospholipids, ceramides, and gangliosides in the center of the plaques in tgArcSwe mice compared with tgSwe mice (Figure S5). Further, 11 of these top 15 discriminative species were observed in the top 15 VIP for OPLS-DA of the average plaque data (Figure S3F). Among the enriched species, LPI (18:0), PI (38:4), and PA (38:4) showed high VIP scores > 1.8. In contrast, Aβ1–42 showed a higher level of enrichment in the plaque center in tgSwe mice. Moreover, the distribution of LPI (18:0) and PI (38:4) showed opposite intra-plaque distribution patterns between the two AD mouse models. In tgArcSwe mice, LPI (18:0) and PI (38:4) displayed a gradient signal with the highest intensity in the center of the plaque. In contrast, LPI (18:0) and PI (38:4) were depleted at the plaque center but accumulated in the periphery of the plaque in tgSwe mice (Figure 3Dii–iii).

This highlights the importance of considering intra-plaque heterogeneity when performing ROI analysis. Specifically, these differential partitioning patterns across plaque ROI would be lost when solely considering the whole plaque data. Further, this highlights the relevance of using structure-sensitive probes to annotate plaque heterogeneity. Common plaque staining tools such as silver staining, thioflavin, or IHC would not permit to outline differences in amyloid fibrillation as warranted by the LCO approach used here.

### Single Plaque Correlation Reveals Lipid–Amyloid Interplays Associated with Aβ Plaque Growth

The first set of multivariate analyses allowed information about plaque-associated lipid and peptide patterns to be obtained on the group level. To further probe the observed lipid–amyloid interplays across individual plaques, correlation analysis was performed for all the correlative lipid and amyloid patterns of the individual plaque features.

**Figure 4:**
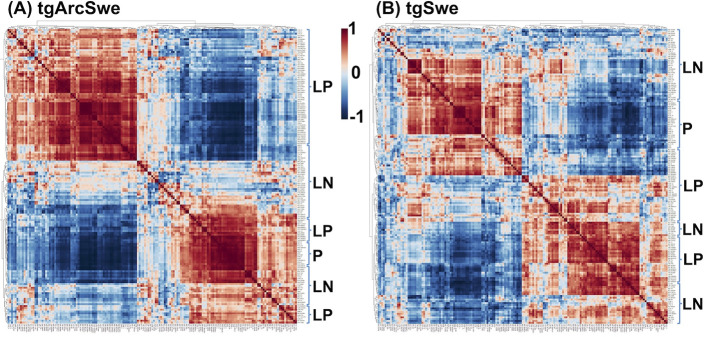
Correlation analysis of plaque center ROI lipid and peptide MSI data. (A) tgArcSwe and (B) tgSwe AD mouse models. Heatmap plots represent the correlation coefficient matrix, reflecting Pearson’s correlation coefficients obtained for multimodal plaque signatures (PCC, intensity scale) including lipids detected in the positive-(LP) and negative ion mode (LN) and amyloid peptides (Aβ).

For this, correlative lipid and peptide MSI imaging data generated from the plaque center (Figure 4) and whole plaque (Figure S6) were analyzed across all the plaques detected within each respective model, resulting in distinct correlation heatmap patterns (Figure 4). Our present data as well as previous studies identified Aβ1–40 to be the prominent Aβ species in plaques observed in tgArcSwe and tgSwe mice.^11,17,35^ We, therefore, compared the top lipid variables that correlated with Aβ1–40 between the two mouse models to get a deeper insights into the lipid–amyloid interplay patterns.

From the correlation analyses, we identified the top 15 lipids that correlated with Aβ1–40 in the plaque center in tgSwe mice, including sphingolipids (*m*/z 616.5 CerP d34:1, *m*/*z* 1544.9 GM1), phosphorserines (PSs) (*m*/*z* 806.5 PS 38:6, *m*/*z* 804.5 PS 38:7, *m*/*z* 822.5 PS 39:5), LPEs (*m*/*z* 436.3 LPE O-16:1, *m*/*z* 462.3 LPE 17:2, *m*/*z* 478.3 LPE 18:1), PIs (*m*/*z* 599.3 LPI (18:0) and *m*/*z* 885.5 PI (38:4)) (all neg. ion mode lipids) and LPCs (*m*/*z* 562.3 LPC 18:0, *m*/*z* 544.3 LPC 20:4, *m*/*z* 568.3 LPC 22:6, *m*/*z* 522.4 LPC 18:1, *m*/*z* 496.3 LPC 16:0) (all pos. ion mode lipids). In contrast, the top 15 lipids that correlated with Aβ1–40 in the plaque center in tgSwe mice included sphingolipids (*m*/*z* 1544.9 GM1, *m*/*z* 715.6 PE-Cer 38:1, *m*/*z* 713.6 PE-Cer 38:2, *m*/*z* 644.5 CerP d36:1, *m*/*z* 642.5 CerP d36:2, *m*/*z* 616.5 CerP d34:1), *m*/*z* 478.3 LPE (18:1), PSs (*m*/*z* 804.5 PS 38:7, *m*/*z* 806.5 PS 38:6, *m*/*z* 822.5 Ps 39:5, *m*/*z* 828.5 PS 40:9) (neg. lipids), and LPCs (*m*/*z* 522.4 LPC 18:1, *m*/*z* 496.3 LPC 16:0), *m*/*z* 720.5 PS (O-32:1), and sphingomyelin *m*/*z* 769.6 SM (d36:1) (pos. lipids) (Figure S7B,D). These differences in the lipid content in plaque centers and whole plaques were associated with the intra-plaque heterogeneity observed for amyloid deposits in tgSwe mice but not for tgArcSwe mice. Consequently, for tgArcSwe mice, the top 15 lipid species correlated with Aβ1–40 in the whole plaque ROI were the same lipid species obsrved to correlate with Aβ1–40 in the plaque center ROI. These included sphingolipids (*m*/*z* 1544.9 GM1, *m*/*z* 715.6 PE-Cer 38:1, *m*/*z* 644.5 CerP d36:1, *m*/*z* 670.5 CerP d38:2), *m*/*z* 435.3 LPA (18:1), LPEs (*m*/*z* 436.3 LPE O-16:1, *m*/*z* 464.3 LPE O-18:1), *m*/*z* 722.5 PE (P-36:4), *m*/*z* 828.5 PS (40:9), *m*/*z* 599.3 LPI (18:0), *m*/*z* 885.5 PI (38:4) (neg, lipids), and LPCs (*m*/*z* 522.4 LPC 18:1, *m*/*z* 544.3 LPC 20:4, *m*/*z* 496.32 LPC 16:0, *m*/*z* 568.3 LPC 22:6) (pos. lipids) (Figure S7A,C).

**Figure 5:**
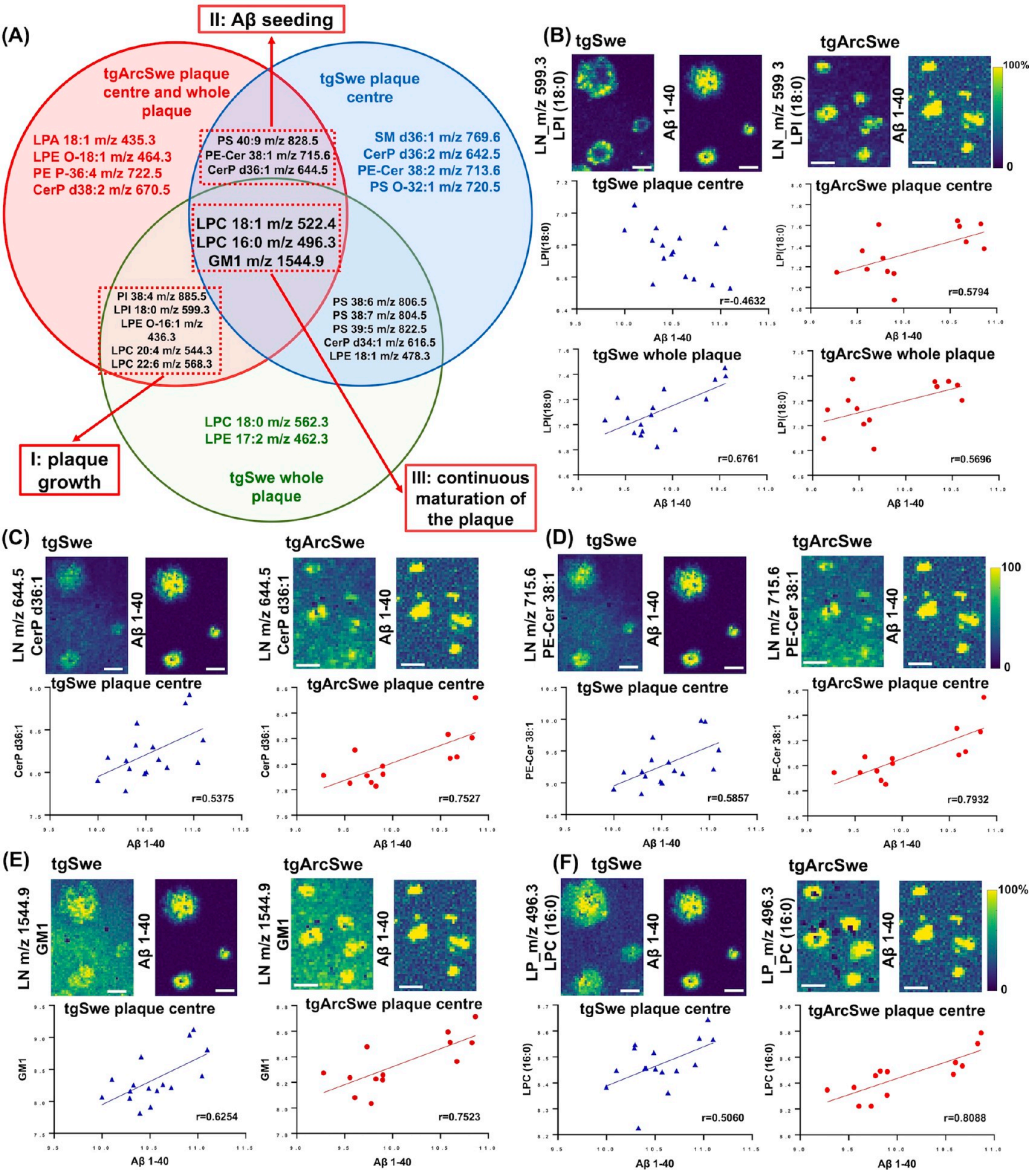
Correlation analyses reveal lipid–amyloid interplays associated with plaque- growth, -fibrillation, and continuous maturation. The Venn diagram (A) displays the top 15 lipids correlated with Aβ1–40 in the whole plaque and plaque center in tgArcSwe and tgSwe mice. Three classes of lipids were implicated in I: plaque growth, II: plaque seeding and increased Aβ fibrillation/core formation, and III: continuous maturation of the plaque. (B) LPI implicated in plaque growth. (C, D) CerP (d36:1) and PE-Cer (38:1) implicated in Aβ seeding/fibrillation. (E, F) GM1 and LPC (16:0) implicated in continuous maturation of the plaque. Scale bar: 100 μm.

Among those lipid–Aβ correlations, we identified three deposition patterns that were implicated with different plaque structures and plaque heterogeneity, respectively (Figure 5A).

Here, LPI (18:0) (Figure 5B), PI (38:4), LPE (O-16:1), LPC (20:4), and LPC (22:6) correlated with Aβ1–40 at the whole plaque level both in tgSwe and tgArcSwe mice. Meanwhile, these lipids were correlated with Aβ1–40 in the plaque center in tgArcSwe mice, but not in tgSwe mice. Considering the polymorphic plaque structures in tgSwe mice, the peripheral enrichment of these lipids suggests solely passive deposition along with Aβ during plaque growth. This could potentially be due to a cellular response toward amyloid aggregation, such as glial (micro- and astro-glial) recruitment. Indeed, LPI and PI species as found here are likely derived from phosphoinositol biphosphate (PIP2) species, which is a major second messenger in PIK3 signaling that mediates recruitment of protein complexes to membranes.^37^ In addition to this, LPI has been identified as an endogenous ligand of TREM2,^38−40^ a receptor implicated in microglia activation in response to amyloid plaques^40−42^ and where the mutation R47H leads to microglial impairment and is a risk factor of AD.^10,41^

### Lipid Correlates with Plaque Formation and Continuous Maturation

The second lipid–Aβ distribution and correlation pattern comprised lipid species that were solely correlated with mature amyloid structures, i.e., at the plaque core in tgSwe and tgArcSwe. This included CerP (d36:1), PE-Cer (38:1) (Figure 5C,D), and PS (40:9) that correlated with Aβ1–40 in the plaque center, but not in the whole plaque in tgSwe and tgArcSwe mice. The distinct correlation with mature Aβ fibrils suggests their implication in the formation (seeding) of cored/compact deposits and Aβ fibrillation. We and others have previously shown that cored plaques mature at the core over time.^12,43^ While it is still unclear, whether those plaques form as small cores as observed in corresponding knock-in models^13,41^ or through later maturation from diffusion into cored plaques,^12^ the relevance of those core-associated lipids is critical as senile (cored) plaque formation has been suggested indicative of plaque neurotoxicity. Indeed, ceramide activation of protein phosphatases PP2a and PP1 has been reported to be involved in apoptosis induction.^44−46^ Moreover, it was previously reported that both CerP and PE-Cer were proposed to mediate ceramide-induced apoptosis and might suggest a mechanism of cellular defense during toxic Aβ aggregation.^46−48^ Furthermore, those anionic lipids are agonists for TREM2 and can play a role in microglia-mediated amyloid clearance.^39,49,50^

In addition to the above lipid–Aβ correlation patterns, we observed that GM1, LPC (16:0) (Figure 5E,F), and LPC (18:1) correlated with Aβ1–40 both in the plaque center and whole plaque in tgSwe mice, as well as in tgArcSwe mice. This finding suggests their implication in continuous plaque growth and amyloid maturation. GM1 is enriched in the CNS and has been reported to be implicated in the regulation of Aβ, with potential implications for AD pathology.^51−56^ In line with this, it was previously reported that increased GM1 and GM2 were found in lipid rafts isolated from the frontal- and temporal cerebral cortex of AD individuals.^53,57^ Herein, from whole plaque to plaque center, GM1 correlated with Aβ1–40 in both AD mouse models. This suggests its implication in continuous Aβ deposition (plaque growth) and maturation (i.e., Aβ fibrillation). Indeed, GM1 was reported to be implicated in AD pathology by binding to Aβ and further promoting Aβ aggregation.^51,52,55^ Moreover, it was recently reported that double-layered structures of Aβ assemblies on GM1-containing membranes catalytically promote fibrillation. All of the above findings along with our results, therefore, support the evidence that GM1 plays a critical role in continuous amyloid maturation from initial plaque formation through plaque growth and maturation.

In addition, LPC (16:0) and LPC (18:1) also showed a strong correlation with Aβ1–40 both at the plaque center and at the whole plaque level in both tgSwe and tgArcSwe mice. Interestingly, LPC was previously reported to increase Aβ oligomer formation.^58,59^ Further, LPC was implicated in demyelination through its conversion to LPA and pathogenic LPA receptor signaling on oligodendrocytes. Together these observations contextualize the multifaceted role of lysophosphatidylcholine species in both pathogenic Aβ aggregation and Aβ initiated neurodegenerative downstream processes.

In summary, in this study tetramodal correlative chemical imaging was employed to examine the lipid–Aβ interplay at amyloid plaques in two transgenic AD mouse models. The results suggest different roles of various lipid species in amyloid aggregation, including their involvement in Aβ plaque growth, Aβ plaque formation and fibrillation, and continuous plaque maturation. While these first correlative lipid–peptide data provide key insight into the role of lipids in amyloid pathology, the technique holds great potential with respect to delineating the role of various Aβ peptide truncations in plaque polymorphism and interactions of those isoforms with different lipids, respectively. This however requires expanding these analyses toward human AD brain tissue including either biopsies or postmortem brain. This is due to the fact that genetic AD mouse models are based on AD-associated mutations in APP or γ secretases (i.e., corresponding rather to familial AD) and do not fully recapitulate human pathology with respect to plaque morphology, Aβ truncation profile, neurodegeneration, cellular response, and co-proteinpathologies, mainly Tau.

## Conclusions

Together, we present a chemical imaging and data analysis strategy for multimodal, correlative (MSI + LM) lipid and protein imaging of histological features at 10 μm resolution. We demonstrate the potential of this approach for gaining a deeper insight into mechanisms underlying AD proteinpathology and disease pathogenesis. Consequently, this imaging paradigm holds great potential for expansion toward spatial multiomics of both human AD brain tissues as well as for studying other diseases.

## Supporting Information Available

The data shown in the SI include: optimization of sample preparation for multimodal MALDI MSI (Figure S1); optical images of mouse brain sections showing the measured regions for each animal (Figure S2); multivariate statistical analysis of group- and single plaque-level data for whole plaque/plaque center ROI from tgArcSwe and tgSwe mice (Figure S3); top loadings for multivariate statistical analyses of trimodal MSI data of whole plaque ROI (Figure S4); top loadings for multivariate statistical analyses of trimodal MSI data for plaque center ROI (Figure S5); correlation analysis of whole plaque ROI lipid- and peptide MSI data (Figure S6); correlation analysis of plaque center ROI lipid and peptide MSI data (Figure S7); OPLS-DA model characteristics (Table S1) (PDF)
