# Supplementary material for: Tetramodal Chemical Imaging Delineates the Lipid–Amyloid Peptide Interplay at Single Plaques in Transgenic Alzheimer’s Disease Models
Source: Anal Chem. 2023 Mar 1;95(10):4692–702. doi: 10.1021/acs.analchem.2c05302 (PMC10018455; doi:10.1021/acs.analchem.2c05302)
Supplement: Supplementary file 1 — ac2c05302_si_001.pdf [file ac2c05302_si_001.pdf]

# Supplementary Information

# Tetramodal Chemical Imaging Delineates the Lipid-Amyloid Peptide Interplay at Single Plaques in Transgenic Alzheimer's Disease Models

Junyue Ge[1], Srinivas Koutarapu[1], Durga Jha[1], Maciej Dulewicz[1], Henrik Zetterberg[1-5], Kaj Blennow[1,2] and Jörg Hanrieder [1,2,3]*

*1 Department of Psychiatry and Neurochemistry, Sahlgrenska Academy at the University of Gothenburg, Mölndal Hospital, House V3, SE-431 80 Mölndal, Sweden*

*2 Clinical Neurochemistry Laboratory, Sahlgrenska University Hospital, Mölndal Hospital, House V3, SE-431 80 Mölndal, Sweden*

*3 Department of Neurodegenerative Disease, Queen Square Institute of Neurology, University College London, London WC1N 3BG, United Kingdom*

*4 UK Dementia Research Institute at University College London, Queen Square, London WC1N 3BG, United Kingdom*

*5 Hong Kong Center for Neurodegenerativa Diseases, Hong Kong, China*

* Correspondence: Dr Jörg Hanrieder,

E-mail: jh@gu.se

Table of content

Supplementary Information Figures S1-S7

Supplementary Information Table S1

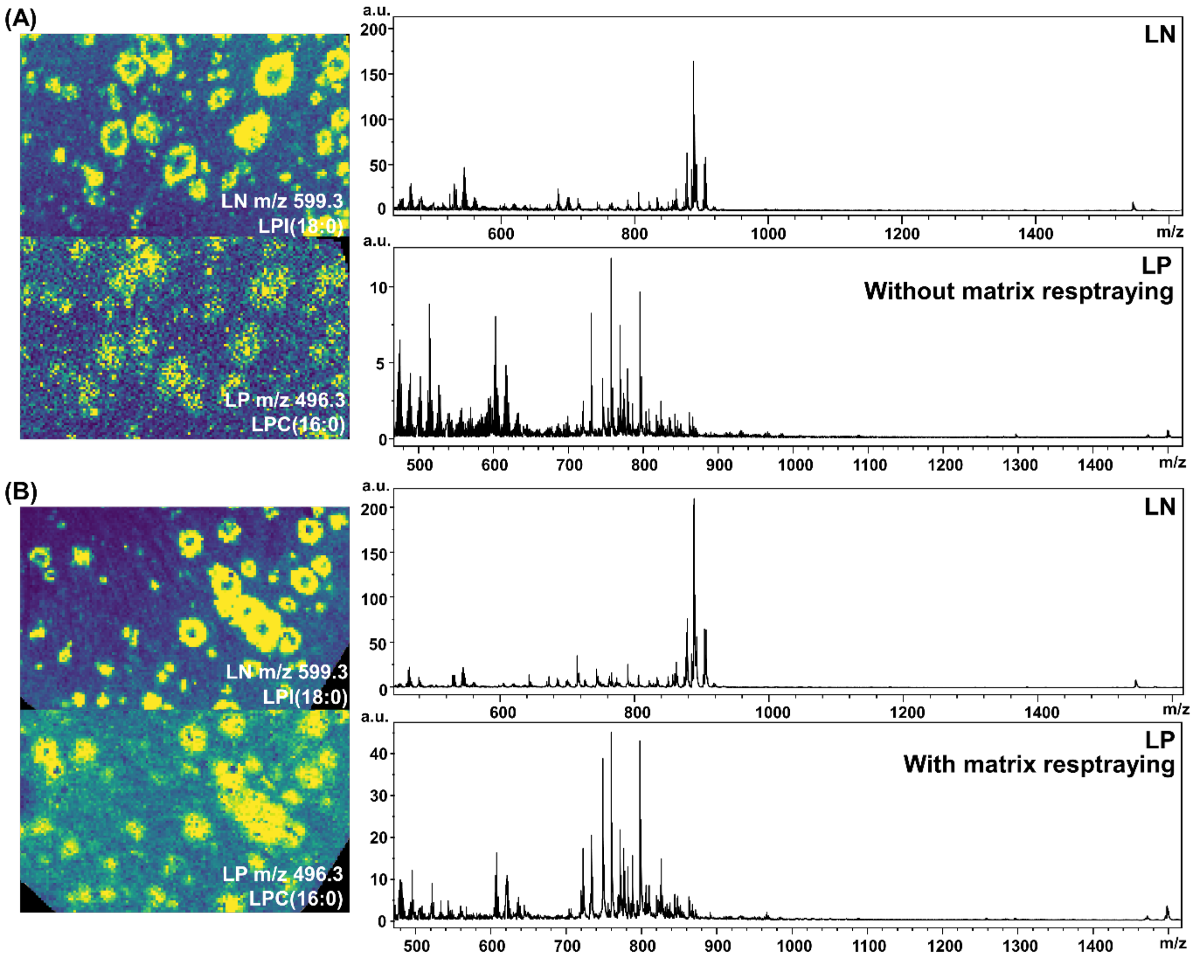


**Figure S1. Optimization of dual polarity MALDI MSI of lipids on the same measured region in the frontal cortex in mouse brain sections.** (A) single ion images and overall spectra of negative and positive MALDI MSI of lipids. The imaging run under positive mode was performed without matrix re-application. (B) single ion images and overall spectra of negative and positive MALDI MSI of lipids. The imaging run under positive mode was performed with matrix re-application.

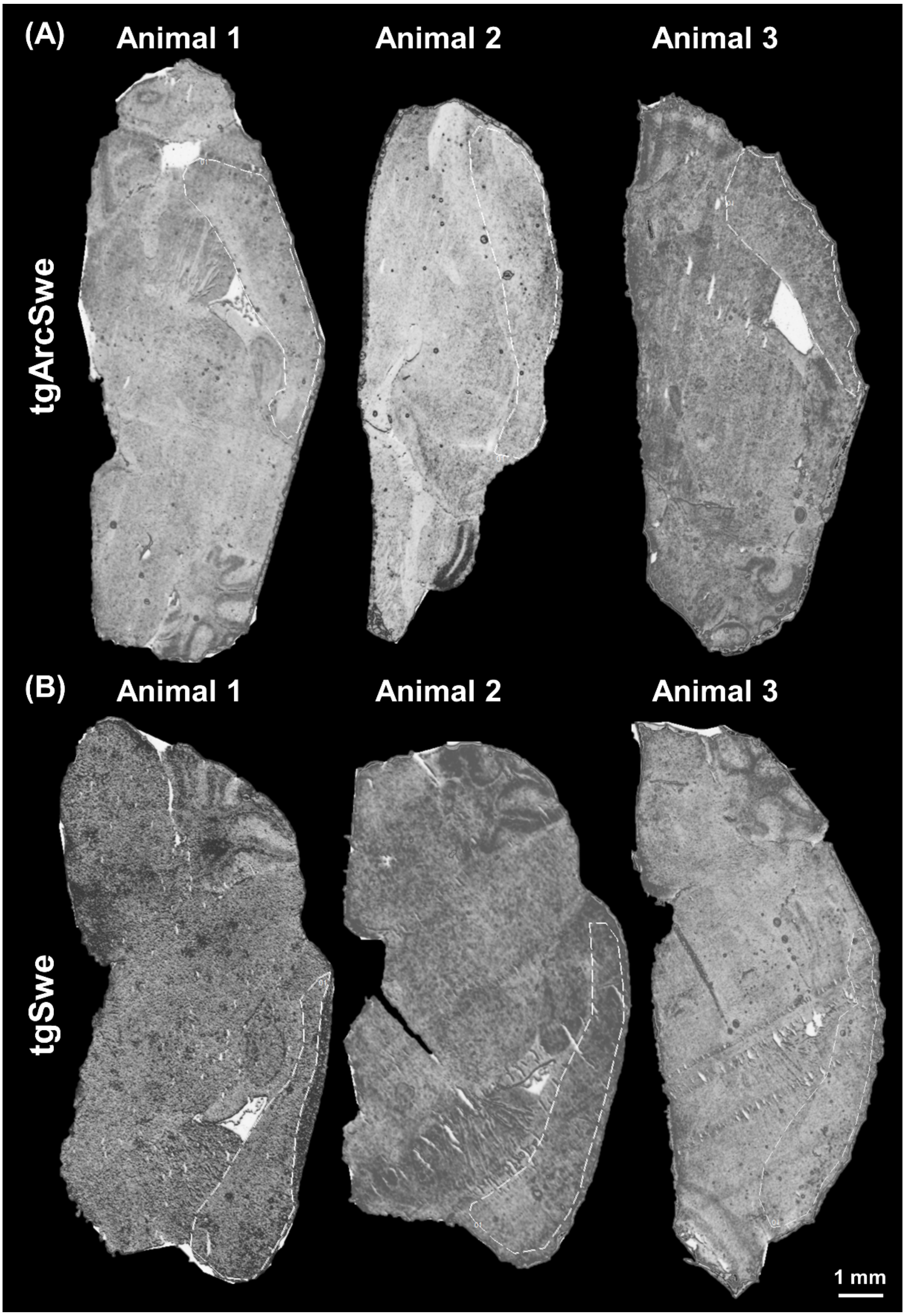


**Figure S2. The optical images of mouse brain sections showing measured regions.** (A) the optical images from tgArcSwe mice and (B) the optical images from tgSwe mice.

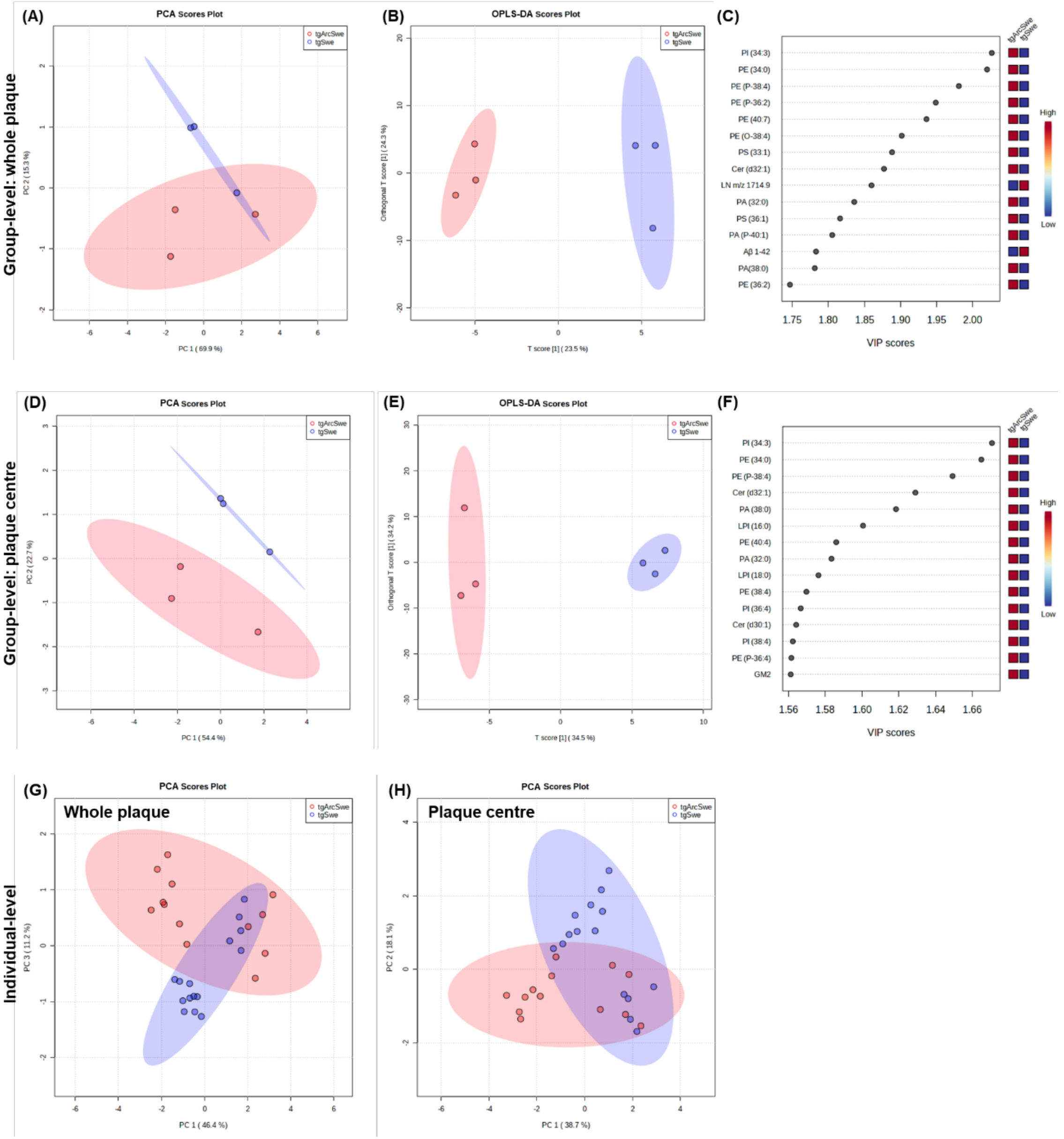


**Figure S3. Multivariate statistical analysis of group- and individual-level data of whole plaque/plaque centre from tgArcSwe and tgSwe mice.** (A) PCA and (B) OPLS-DA score plots showed good separations of the groups based on group-level data of whole plaque, (C) VIP scores plot described top 15 variates differentiating two groups. (D) PCA and (E) OPLS-DA score plots showed good separations of the groups based on group-level data of whole plaque, (F) VIP scores plot described top 15 variates differentiating two groups. PCA scores plot for the individual-level data of whole plaque (G) and plaque centre (H) from two groups.

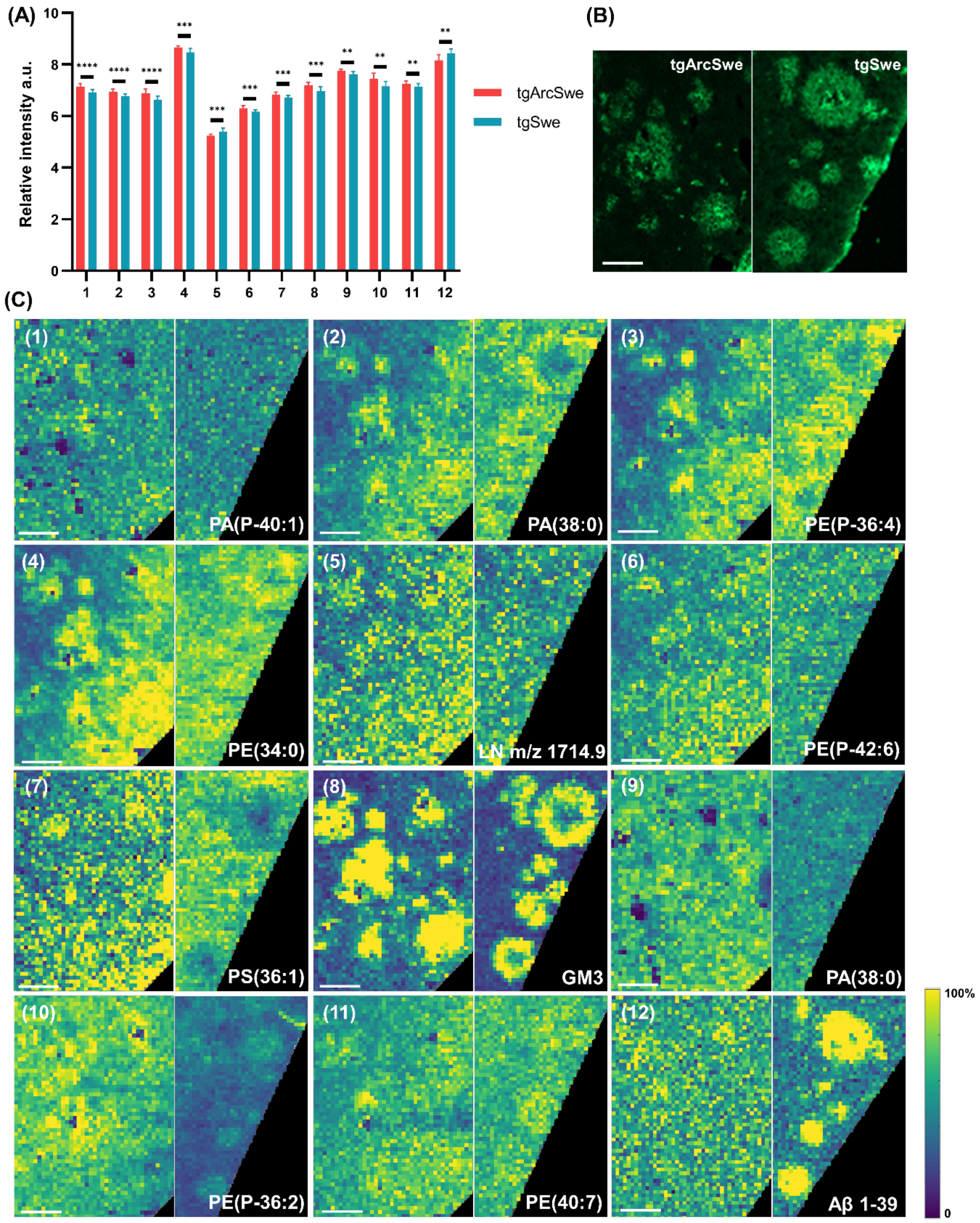


**Figure S4. Multivariate statistical analyses of trimodal MSI data reveal different levels of lipid enrichment in the whole plaques in tgArcSwe and tgSwe AD mouse models.** (A) Statistical analysis of top 4 to 15 lipids differentiating two populations in tgArcSwe and tgSwe AD mouse models. (B) LCO images and (C) single ion images of lipids differentiating two populations in tgArcSwe (left) and tgSwe (right) AD mouse models.

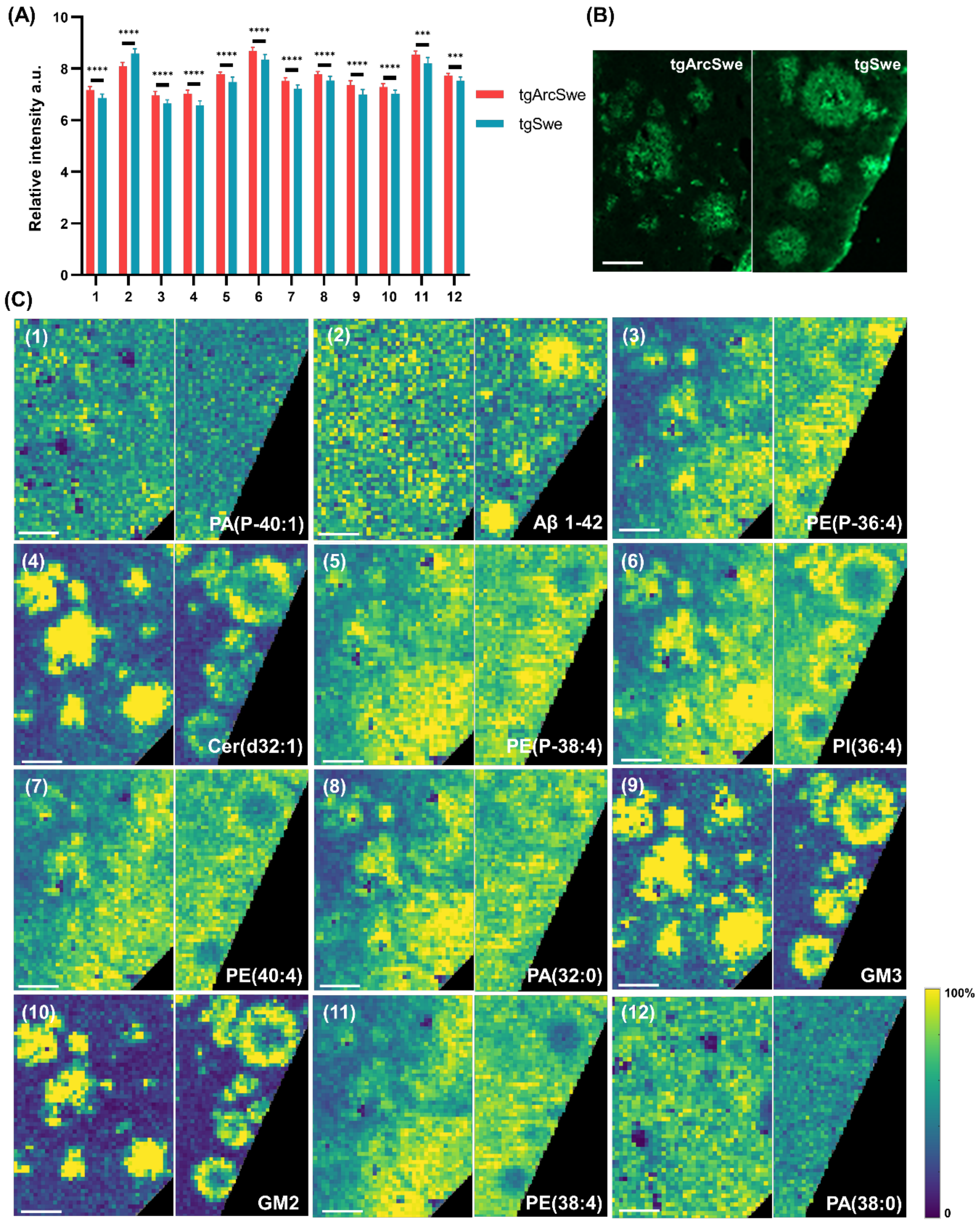


**Figure S5. Multivariate statistical analyses of trimodal MSI data reveal different levels of lipid enrichment in the centre of the plaque in tgArcSwe and tgSwe AD mouse models.** (A) Statistical analysis of top 4 to 15 lipids differentiating two populations in tgArcSwe and tgSwe AD mouse models. (B) LCO images and (C) single ion images of lipids differentiating two populations in tgArcSwe (left) and tgSwe (right) AD mouse models.

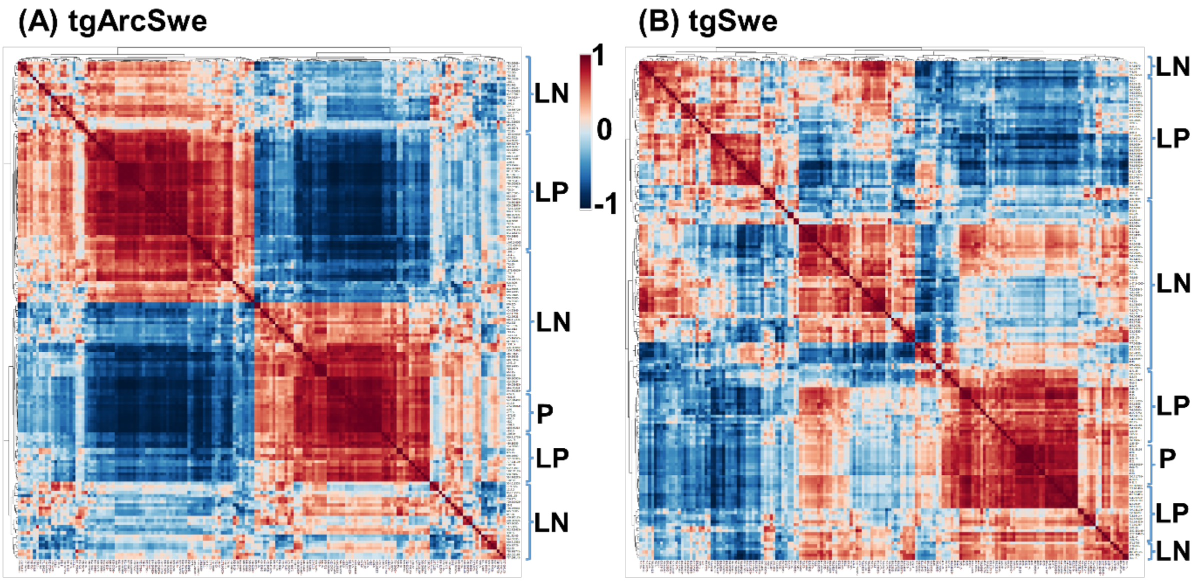


**Figure S6. Correlation analysis of whole plaque ROI lipid and peptide MSI data.** (A) tgArcSwe and (B) tgSwe AD mouse models. Heatmap plots represent the correlation coefficient matrix, reflecting Pearson's correlation coefficients (PCC).

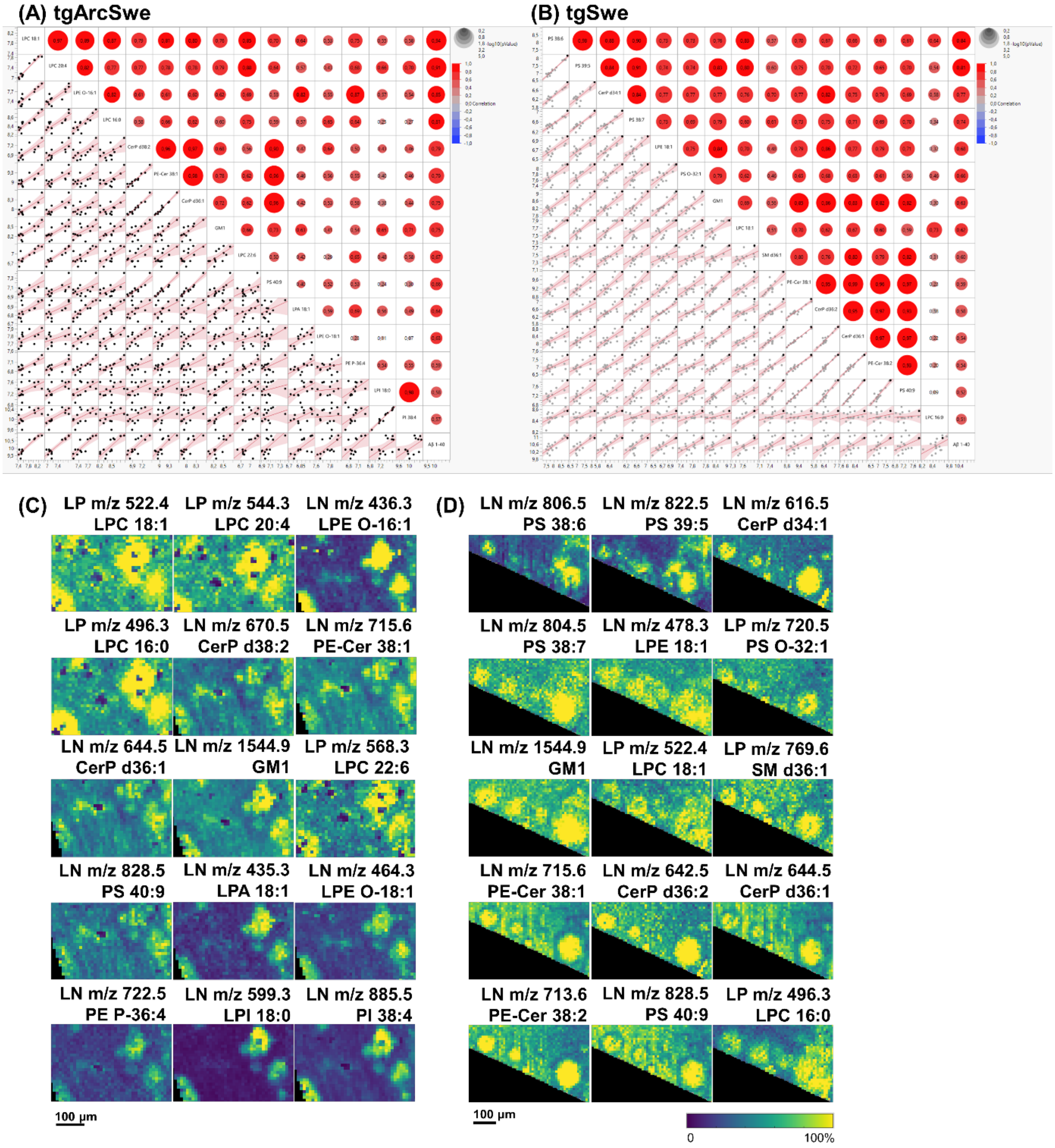


**Figure S7. Correlation analysis of plaque ROI lipid and peptide MSI data and single ion images.** (A, B) Correlation plots represent the top 15 variates of lipids correlating with Aβ 1-40 in (A) tgArcSwe and (B) tgSwe mouse models, reflecting Pearson's correlation coefficients (PCC). (C, D) Single ion images of top variates of lipids correlated with Aβ 1-40 in plaque centre in (C) tgArcSwe and (D) tgSwe AD mouse models.

**Table S1. OPLS-DA model characteristics. R2X, R2Y represents the model interpretation rate; Q2 indicates the model predictive ability; R2Y and Q2 closer to 1 indicates that the model more stable and reliable.**

| | Model | R2X | R2Y | Q2 |
|---|---|---|---|---|
| **Whole plaque (group-level)** | OPLS-DA | 0.235 | 0.969 | 0.313 |
| **Plaque centre (group-level)** | OPLS-DA | 0.345 | 0.928 | 0.670 |
| **Whole plaque (individual-level)** | OPLS-DA | 0.107 | 0.843 | 0.700 |
| **Plaque centre (individual-level)** | OPLS-DA | 0.209 | 0.769 | 0.695 |
